# Brain Magnetic Spectroscopy Imaging and Hereditary Spastic Paraplegia: A Focused Systematic Review on Current Landmarks and Future Perspectives

**DOI:** 10.3389/fneur.2020.00515

**Published:** 2020-07-14

**Authors:** Marinela Vavla, Domenico Montanaro, Silvia Pizzighello, Francesca Frijia, Filippo Arrigoni, Alessandra Baratto, Gianluca Piccoli, Gabriella Paparella, Andrea Martinuzzi

**Affiliations:** ^1^SOS Neuromotor Unit, Department of Pieve di Soligo, Scientific Institute, IRCCS E. Medea, Treviso, Italy; ^2^SOS Neuromotor Unit, Department of Conegliano, Scientific Institute, IRCCS E. Medea, Treviso, Italy; ^3^Department of Women's and Children's Health, University of Padova, Padua, Italy; ^4^U.O.C. Risonanza Magnetica Specialistica e Neuroradiologia, Fondazione CNR/Regione Toscana G. Monasterio, Pisa, Italy; ^5^U.O.C Bioengineering and Clinical Technology, Fondazione CNR/Regione Toscana G. Monasterio, Pisa, Italy; ^6^Neuroimaging Lab, Scientific Institute, IRCCS E. Medea, Bosisio Parini, Italy; ^7^Department of Radiology S. Maria dei Battuti Hospital-Conegliano, Treviso, Italy

**Keywords:** magnetic resonance spectroscopy (MRS), hereditary spastic paraplegia (HSP), systematic review, brain metabolites, neurodegeneration, SPG

## Abstract

Magnetic resonance spectroscopy (MRS) is a non-invasive neuroimaging technique used to investigate *in vivo* brain metabolites. MRS could provide a sensitive tool for the study of hereditary spastic paraplegia (HSP) by helping to unveil the underlying biochemical mechanisms and monitoring response to treatment. This focused systematic review aimed to summarize the brain metabolite findings in studies performed in genetically determined HSP. The second aim was to provide a critical analysis and recommendations for well-designed protocols for future studies. Fourteen MRS studies have been analyzed with overall 61 HSP patients, falling within a wide range of age at onset, disease duration, and age at the MRS scan, including children and adults. The genetic diagnosis included several subtypes (SPG2/3/4/5/10/11/28/31/54). SPG11 and SPG54 have been more frequently investigated. The MRS methodology included different MR field strength, not easily comparable spectra areas varying from whole brain to various cortical areas, brain stem and cerebellum sampling. No consistency in disease severity and other outcome measures was observed. The main MRS findings corresponded to the white matter metabolite abnormalities in the corticospinal tracts. In summary, this focused review provides insights on the current knowledge of brain metabolites in HSP and, in particular, in SPG11 and SPG54. Despite the inhomogeneity of the studies to date reported, brain metabolites as assessed by MRS could represent potentially useful diagnostic markers and prognostic indicators of disease progression in HSP. Specific recommendations regarding the MRS technical protocol, CNS area sampling, study design, and applicability of findings are given.

## Introduction

Brain magnetic resonance spectroscopy (MRS) is a non-invasive imaging technique useful to provide quantitative measures of specific brain metabolites (1). The main metabolites sampled can reflect neuronal integrity [N-acetyl-aspartate (NAA)], membrane turnover [choline (Cho)], glial cell integrity [myo-inositol (mI)], energy metabolism [creatine (Cr)], anaerobic glycolysis products [lactate (Lac)], products of brain damage [lipids (Lip)], and less frequent others. Brain MRS techniques are applied in several CNS disorders such as ischemic lesions, tumors, infections, demyelinating conditions, epilepsy, and neurodegenerative disorders with the intent to study basic physiology and disease processes and measure treatment outcomes (2). To date, the spinal cord MRS application is feasible but complex and still controversial due to several anatomical and functional confounding factors (2, 3).

Hereditary spastic paraplegias (HSPs) are a group of rare heterogeneous disorders that affect the cortical spinal tracts (CSTs) presenting clinically with muscle weakness and spasticity in the lower limbs (4). The HSP pathogenic mechanisms include myelination disorders, axonal transport and pathfinding, lipid metabolism, and mitochondrial (dys)function (5), sustained by a wide genetic heterogeneity. Given this puzzle-like picture of the HSP neuropathological mechanisms, there are many difficulties in the development of treatment options and clinical trial design. The search for biomarkers is still incomplete despite the use of different tools. We have previously investigated the baseline intersection of diffusion tensor imaging (DTI) indexes in genetically determined HSP patients (6) and recently the longitudinal variation of the retinal nerve fiber layer thickness (7).

Detecting biochemical changes in HSP could be a helpful hand when searching for diagnostic tools and surrogate biomarkers. Although MRS is laborious and time-consuming and there is still no standard defined protocol, this technique could take the lion's share in providing the profile of *in vivo* biochemical changes in HSP, unraveling the involvement of motor and non-motor areas and providing insights on genotype-specific neuropathological substrates. This focused review aims to give an overview of all the MRS studies that investigated brain metabolites in genetically determined HSP patients.

The first aim is to summarize the metabolic changes and their cerebral distribution in HSP as described in the scientific reports. In particular, we reported the MRS findings in SPG11 and SPG54 genotypes that are the most frequently analyzed SPG types so far. The second aim is to present a critical discussion regarding the use of MRS as a diagnostic tool in HSP either as a first-level examination or in the differential diagnosis process. The overall findings of this review will be helpful to highlight any potential role of MRS in providing biomarkers for the study of disease progression, clinical trial design, and validation of new treatments.

## Methods

This systematic review was written in accordance to the PRISMA statement (8). The *Methods* section is presented in the Supplementary Material and Supplementary Figure 1.

## Results

### Demographic Characteristics

The studies included in this review were published in a time lapse of 13 years. Table 1A shows the geographic distribution of the countries to which the first authors were affiliated. Studies considered in this review fell into two main categories (22) as clinical research (case reports, case series) and epidemiological research (case control studies), consisting of 10 and 4 studies, respectively. Only two studies included a follow-up of 4 and 5 years (13, 21). All studies except one described data from a single SPG type. Although Martinuzzi et al. (6) presented the biggest HSP sample investigated with MRS so far, it included a heterogeneous group of six different SPG types. SPG11 and SPG54 subtypes were the most represented ones. An overall number of 61 HSP patients were reported in all the reviewed records, with a gender distribution of 31 females and 30 males. Patients' ages at MRS scan ranged from 3 to 69 years. Samples varied from one single patient to a maximum of 22 patients. Only four studies reported healthy control (HC) groups undergoing the MRS scan (overall 42 subjects) (6, 11, 18, 20), all matching in size the HSP group. Disease severity was assessed with the specific Spastic Paraplegia Rating Scale (SPRS) (23) in a few studies (6, 9, 11). Additional results on the flowchart of the search strategies and clinical data and quality of the studies can be found in Supplementary Material and Results and Supplementary Figures 1, 2.

**Table 1A:** Demographic characteristics of the studies and patients.

**Y**	**References**	**Journal**	**First author's country of affiliation**	**Category of clinical research**	**Genetic diagnosis**	**HSP sample size**	**F (%)**	**Age at MRS (years)**	**HC sample size**
2019	Nicita et al. (9)	*Journal of Neurology*	Italy	Clinical research: single case report	SPG54	1	0 (0)	56	0
2019	Thabet et al. (10)	*Archives of Disease in Childhood*	Saudi Arabia	Clinical research: single case report	SPG54	1	1 (100)	4	0
2017	Schneider-Gold et al. (11)	*Journal of Neurological Sciences*	Germany	Epidemiological research: case control study	SPG11	2	2 (100)	22 (twins)	2
2016	Fraidakis et al. (12)	*Neurodegenerative Diseases*	Greece	Clinical research: single case report	SPG11	1	1 (100)	30	0
2016	Martinuzzi et al. (6)	*PlosOne*	Italy	Epidemiological research: case control study	SPG4 SPG3 SPG5 SPG10, SPG11, SPG31	22	10 (45)	Mean 41.5 (range 9–69)	22
2015	Lossos et al. (13)	*Brain*	Israel	Clinical research: case reports (4 years follow up)	SPG75	3	1 (33)	17, 22, 23 (baseline)	0
2014	Doi et al. (14)	*Scientific Reports*	Japan	Clinical research: single case report	SPG54	1	1 (100)	69	0
2014	Liguori et al. (15)	*Journal of Neurology*	Italy	Clinical research: case reports	SPG28	2	0 (0)	35, n.r.	0
2014	Roos et al. (16)	*Acta Neurologica Scandinavica*	Denmark	Clinical research: case reports	SPG5	2	2 (100)	26,29	0
2012	Schuurs-Hoeijmakers et al. (17)	*The American Journal of Human Genetics*	Netherland	Clinical research: case series	SPG54	5	2 (40)	3, 5, 7, 10, 30	0
2011	Stromillo et al. (18)	*Journal of Neurology*	Italy	Epidemiological research: case control study	SPG11	10	6 (60)	Mean 29.7 ± 8 (range 18-46)	10
2010	Svenstrup et al. (19)	*Journal of Neurology, Neurosurgery and Psychiatry*	Denmark	Clinical research: case reports	SPG2	2	0 (0)	28 and 20	0
2009	Erichsen et al. (20)	*Journal of Neurological Sciences*	Norway	Epidemiological research: case control study	SPG4	8	4 (50)	Mean 48.4 ± 7	8
2006	Dreha-Kulaczewski et al. (21)	*Neuroradiology*	Germany	Clinical research: single case report (5 years follow up)	SPG11	1	1 (100)	20	0

*The studies are listed chronologically from the most recent year of publication*.

*F, female; Ref., reference; HSP, hereditary spastic paraplegia; MRS, magnetic resonance spectroscopy; HC, healthy control*.

### General Magnetic Resonance Spectroscopy Findings in All the Studies

The spectra were sampled in the white matter (WM)/gray matter (GM) structures of the following brain regions: frontal, parietal and occipital cerebral cortex, centrum semiovale, splenium of corpus callosum (CC), corona radiata, basal ganglia (BG), thalamus, periventricular WM, cerebellar GM, precentral WM/GM, and brain stem (Table 1B). One study investigated metabolite distribution in the cerebrospinal fluid (CSF) of the lateral ventricles (15). All metabolites were evaluated as the ratio to Cr, Cho, or mI or as absolute concentrations depending on the specific software applied (LcModel Dekra) (12, 19).

**Table 1B:** MRS findings in the studies.

**References**	**MRS metabolite analysis**	**MR field strength**	**Localization of the Voxel**	**NAA (2.01 ppm)**	**Cho (3.23 ppm)**	**Creatine (3.93 ppm)**	**mI (3.58 ppm)**	**lipids (0.9 and 1.4 ppm)**	**Lactate (1,32–1.36 ppm)**	**Clinical and para-clinical correlations**	**Other neuroimaging techniques**
Nicita et al. (9)	Lipids	1.5T	BG, Thalamus	n.r.	n.r.	n.r.	n.r.	n.d.	n.d.	n.r.	n.r.
Thabet et al. (10)	All metabolites	n.r.	BG	normal	normal	normal	normal	Present	n.d.	n.r.	n.r.
Schneider-Gold et al. (11)	NAA/Cr, NAA/Cho, NAA/mI and Cho/Cr	3T	CC splenium, GM cerebellum	CC: moderate reduction NAA/Cr, NAA/mI (*n* = 1)	n.r.	n.r.	n.r.	n.d.	n.d.	n.r.	DTI
Fraidakis et al. (12)	All metabolites (NAA/Cr, Cho/Cr, mI/Cr)	3T	L parietal WM, R centrum semiovale, R frontal cortex, Brain stem,	reduction in L parietal WM and R centrum semiovale	reduction in L parietal WM	reduction in L parietal WM	n.r.	n.d.	n.d.	n.r.	DTI
Martinuzzi et al. (6)	All metabolites	1.5T	Precentral WM/GM.	normal	normal	normal	normal	n.d.	n.d.	n.r.	DTI
Lossos et al. (13)	NAA/Cr, Cho/Cr	n.r.	centrum semiovale	increased	Increase Cho/Cr, Cho/NAA	n.r.	n.r.	n.d.	n.d.	n.r.	n.r.
Doi et al. (14)	Lipids	3T	L thalamus, BG	n.r.	n.r.	n.r.	n.r.	Present	n.d.	n.r.	n.r.
Liguori et al. (15)	All metabolites	n.r.	Brain cortex, CSF in lateral ventricles	normal	normal	normal	normal	n.d.	Present (n = 1): mild accumulation in CSF in lateral ventricles	n.r.	muscle MRS
Roos et al. (16)	All metabolites	n.r.	O-P WM	n. r.	n.r.	n.r.	increased mI/Cr	n.d.	n.d.	n.r.	n.r.
Schuurs-Hoeijmakers et al. (17)	Lipids	1.5T	BG, thalamus	n. r.	n.r.	n.r.	n.r.	Present	n.d.	n.r.	n.r.
Stromillo et al. (18)	All metabolites	1.5T	CC, superior regions of corona radiata, periventricular WM	Reduced NAA/Cr in Corona radiata and periventricular WM	normal	n.r.	n.r.	n.d.	n.d.	Correlation of reduced NAA/Cr in corona radiata with increased disease scores	n.r.
Svenstrup et al. (19)	All metabolites	1.5T	O-F WM, Mid-occipital GM, centrum semiovale	Reduced in WM in one patient and normal in the other	WM: increasing (*n* = 1)	WM: increasing (*n* = 1), GM: increasing (*n* = 1)	WM: increasing	n.d.	n.d.	n.r.	18F-FDG-PET
Erichsen et al. (20)	All metabolites (NAA/Cr, NAA/Cho, Cho/Cr, mI/Cr)	1.5T	L F WM lateral to genus of CC, central sulcus in precentral gyrus of motor cortex GM	Reduced (not signific.) in F WM and motor cortex.	Motor cortex: reduction	n.r.	F WM and motor cortex: increasing (but not signific.)	n.d.	n.d.	Cho/Cr reduction correlated with age-related verbal-learning short-term memory	n.r.
Dreha-Kulaczewski et al. (21)	All metabolites (absolute concentration)	T_0_: 2T only MRS; T_1_: 3T MRS, DTI.	F and P-O WM, Paramedian parietal GM	Reduced in F and P-O WM	Reduced in F and P-O WM at follow-up	Reduced in F WM	Reduced in F WM, stable P-O WM	n.d.	Increased in P-O WM.	n.r.	DTI

*n.r., not reported; n.d, not detected; F, frontal; P, parietal; O, occipital; BG, basal ganglia; CC, corpus callosum; GM, gray matter; WM, white matter; L, left; R, right; NAA, N-acetyl-aspartate; Cho, choline; Cr, creatine; mI, myoinositol; Lip, lipids; Lac, lactate; DTI, diffusion tensor imaging; MRS, magnetic resonance spectroscopy; ppm, parts per million; T0, at baseline; T1, at follow-up*.

Normal NAA values were detected in few single reports, respectively in BG, in the CSF of the lateral ventricles (10) and in a non-specified region of the brain cortex (15). NAA metabolite was reported widely reduced in most of the studies, in the WM areas (splenium of CC, corona radiata, centrum semiovale and periventricular regions), and also in the cortical regions (parietal, parietal-occipital and frontal) (11, 12, 18–21). Only one single study reported an increase of NAA/Cr in the centrum semiovale (13).

Cho levels were analyzed as absolute concentration and ratio to Cr. Cho levels were reported to be normal in BG, brain cortex, CSF of the lateral ventricles, CC, corona radiata and in the periventricular WM (10, 15, 18). Cho was reduced in frontal, parietal and parietal-occipital WM and in the cortical motor areas (12, 20, 21). An increase of Cho was registered in the centrum semiovale and in the parietal and occipital WM areas (13, 19).

Cr levels were analyzed as absolute concentration. Two studies reported normal values of Cr in the brain cortex, BG and in the CSF of the lateral ventricles (10, 15). Cr metabolite appeared reduced in parietal and frontal WM (12, 21). One study reported an increase of the Cr levels in the parietal-occipital WM and occipital GM (19).

The mI levels were analyzed as absolute concentration and ratio to Cr. The mI was reported to be normal in the brain cortex, BG and in the lateral ventricles CSF (10, 15). Reduced levels of mI were registered in one single study in the frontal WM and reported stable in the parietal-occipital WM during the follow-up (21). Three studies registered an increase of mI in the frontal (but not significant), parietal-occipital WM, and also in the occipital GM (but not significant) (16, 19, 20).

Presence of lipid peaks was reported in three studies with MRS samplings in the BG and thalamus (10, 14, 17). The other reports did not describe any lipid levels, although the MRS sample frequencies were comprehensive of the lipids range.

The Lac absolute concentrations were reported increased in the parietal and occipital WM and in the lateral ventricles CSF (15, 21).

**Figure 1:**
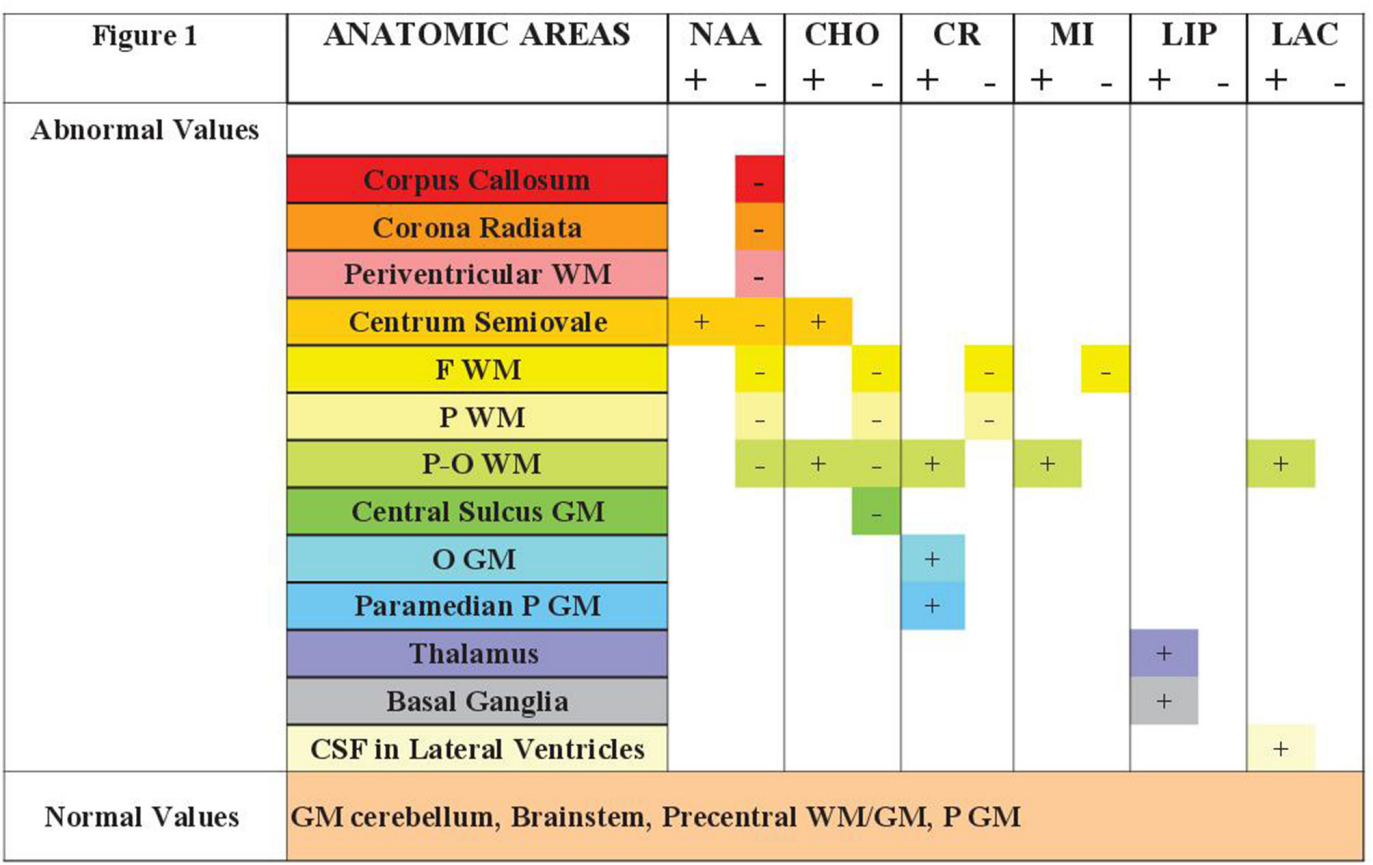
The general findings of brain metabolites variations reported in the MRS studies conducted in HSP and selected for the present review. Presentation of the anatomic regions sampled for spectroscopy and the metabolic pattern compared to healthy controls. NAA, N-acetyl-aspartate; Cho, Choline; mI, myoinositol; Cr, creatine; Lip, lipids; Lac, lactate; WM, white matter; F, frontal; P, parietal; O, occipital; GM, gray matter; CSF, cerebro-spinal fluid; −, reduced; +, increased.

The most relevant findings have been reported in Figure 1.

Two studies reported correlation analysis with clinical variables, such as the inverse correlation of NAA/Cr reduction in the corona radiata with the progression of landmarks of disability and a correlation between Cho/Cr reduction and age-related verbal learning memory tests (18, 20).

### SPG11 Subgroup

Five studies have reported MRS data in SPG11 patients. One study (6) was excluded due to the genetic heterogeneity of the sample. Another study reported longitudinal data but since they used two different MRI fields with 2.0 and 3.0 Tesla (T) scans, the results are difficult to compare (21). Overall, 14 SPG11 patients were studied (10 females) with an age range at the MRS scan of 18–46 years (11, 12, 18, 21). Generally, the NAA metabolite was reduced as NAA or NAA/Cr in the CC (11), centrum semiovale (12), left parietal WM (12), corona radiata (18), in cortical regions (frontal, parietal and occipital) and periventricular WM (18, 21). Cho metabolite was reported either reduced in the parietal, occipital and frontal WM, and in the paramedian GM (12, 21), or normal in areas such as corona radiata, CC and periventricular WM (18). Cr levels were reported reduced in parietal, occipital and frontal cortex and paramedian parietal GM (12, 21). The mI levels were reduced in frontal WM and stable in the parietal and occipital WM (21). Lipids were not reported in any of the SPG11 studies. Lac was reported increased in parietal and occipital WM (21). One study reported correlations between NAA/Cr reduction and GM atrophy in cortex, BG, thalami and damaged WM (18). NAA/Cr reduction correlated with disease severity increase. The common structural findings at MRI of the studies on SPG11 patients consisted in the presence of thin corpus callosum (TCC), WM hyperintensities and brain atrophy. Moreover, DTI indexes were altered (reduced FA, increased MD) in the CC, CST, frontal and occipital WM.

### SPG54 Subgroup

Four studies reported findings in patients with genetic diagnosis of SPG54. These were all case reports, with overall 8 patients (4 females) and age range of 3–69 years (9, 10, 14, 17). No HC groups were included in these studies. Three studies detected presence of lipid metabolites in the BG and thalamus (10, 14, 17). The other metabolites were reported within normal ranges in a single study (10) or not reported at all (9, 14, 17). No correlation analyses were reported. Mostly, in these studies there were descriptions of TCC and WM changes.

### MRS Technical Aspects

All studies applied a single-voxel method with acquisition at short echo time (TE), two applied long TE (14, 17) and one multivoxel acquisition (18). Most of these studies, in addition to cerebral MRS, included brain morphometry assessments and also DTI (9, 11, 12, 21), 18F-FDG-PET (19) and one muscle MRS (15). Erichsen et al. (20) designed a case control study with a single neuroimaging technique (MRS).

The MRS scan field strength varied from 1.5 T to 3.0 T, although four studies failed to report it. One of the follow-up studies used two different magnets for the two time-point acquisitions (21). The metabolites analysis has been reported as either concentration or as ratios (Table 1B) and specific software packages were applied for the analysis (LcModel or Dekra). One study analyzed and reported both concentrations and ratios of metabolites (12). The metabolites NAA, Cho and mI were variably reported as ratios to Cr, Cho and mI. Few studies reported the type of sequence applied, such as Point Resolved SpectroScopy (PRESS) (11, 12, 14, 20) and STimulated Echo Acquisition Mode (STEAM) (21), while other studies failed to indicate it.

## Discussion

Here we present the results of a systematic review on brain MRS findings in patients with genetically-determined HSP. Fourteen articles satisfied the inclusion criteria and only four of them included control groups. Overall, 61 patients have been MRS-investigated with a wide age range and equal gender-ratio. These results should be discussed cautiously since they derive from small samples with limited power and include only two follow-up studies. Therefore, it is fundamental to design longitudinal studies with bigger sample sizes in order to detect real significant changes over time.

The applied heterogeneous clinical, neurophysiological, and neuropsychological outcome measures appeared to have mostly a diagnostic valence. The outcome measures should reflect the specific research questions and be sensitive to typical changes in HSP. Therefore, we suggest that the SPRS score should be included as a clinical measure of disease severity in future studies. The scale has not been validated for infants and small children, but should be included at least when dealing with patients above age six. Among these studies, SPG11 and SPG54 have been specifically investigated in four cohorts each, whereas for other SPG types we could retrieve much sparser literature frequently consisting of single case studies.

SPG11 was characterized by widespread cerebral NAA reduction (splenium CC, corona radiata, centrum semiovale, periventricular, frontal, parietal and occipital WM) and also, combined NAA, Cho and Cr reductions in parietal WM (12, 21). The analysis provides hints of correlation between the brain metabolite levels in SPG11 and disease severity, structural and functional abnormalities. All these SPG11 studies, with concomitant spatacsin mutations involved in axonal stability and transport (12), presented additional findings such as TCC, WM hyperintensities, brain atrophy, and impaired DTI indexes in areas related to CST tracts.

Consistent and overlapping increased lipid peaks were observed in the SPG54 subtype and located in the BG and thalamus. These results could be meaningful from a biochemical prospective, because the gene involved (DDH2) in SPG54 is a key player of phospholipid and fatty acid metabolism (17, 24). Supposedly, the BG and thalamus areas could be further explored in other SPG types that share similar lipid metabolism involvement (e.g., SPG5, SPG28, SPG31, SPG38, SPG39, SPG46, SPG56). These findings could add up to the differential diagnosis between the SPG11 and SPG54. Although TCC is a common finding in these two forms, the lipid peaks detected in BG and thalamus is present only in SPG54. Therefore, the MRS lipid detection could contribute to the differential diagnosis and/or identification of the SPG54 patients that share TCC findings with other SPG types.

Spectra areas selection was broadly localized in the brain WM and GM, and in particular in the cerebral cortex, supratentorial WM, BG, cerebral CST portions and cerebellum. None of these studies included spinal cord MRS. The metabolite impairment distribution was widely spread in many anatomical sites such as the corona radiata, peri-ventricular and frontal-parietal WM. NAA was widely and diffusely reduced in the CNS as reported previously (25). Cho was reduced in parietal WM and corona radiata, but few reports described also increased values (13, 19). The mI resulted normal or increased in all but one study (21). Variations of other specific metabolites, although not routinely sampled, consisted in Lac detection in CSF (15) and lipid presence in BG and thalamus in SPG54 (10, 14, 17).

The different anatomical spectra localization and the non-overlapping anatomical terminology is a limitation that leads to confusion. The selected studies in this review have mainly investigated corona radiata, internal capsule and centrum semiovale, that lead axons to and from the cortex, and also in the interhemispheric commissure fibers, that connect to the various cortical structures (parietal, occipital, frontal). This anatomical choice of spectra areas reflects an attempt to explore the metabolites changes along the CST. We highlight the importance of this choice and recommend future studies of the metabolites in corona radiata, parietal and frontal subcortical WM along the CST regions. The HSP widespread cerebral involvement has perhaps guided many authors to study scattered areas in the brain. Despite the interesting results, the lack of anatomical overlap makes it difficult to generalize the findings between different studies and various SPG forms.

As the MRS technique is time-consuming depending on the number of spectra samples detected, the extension of the sampling spectra in other brain regions should be reasonably considered only for clinical trials or follow-up studies and taking into consideration the general clinical conditions of the patients.

The brain stem and spinal MRS studies are scanty. Thus, in order to assure good quality, spectra regions should be selected far from inhomogeneous interfaces (CSF, bone, air, vascular structures, etc.) as they affect the ability to distinguish the signal of the different metabolites.

Various technical aspects can influence the final results of the MRS sampling. Most of the selected studies were conducted with a short Echo Time (TE). This is usually recommended as it facilitates the detection of many metabolites when compared to long TE ones, that are more suitable to analyze the lipid frequencies or investigate lactate levels. The spectral resolution, defined as the ability to distinguish the various metabolic peaks from each other, is highly dependent on the intensity of the magnetic field. Consequently, the use of 3.0 T equipment, rather than 1.5 T, is recommended. The role of ultrahigh magnetic fields (7.0 T) is not entirely defined. It is strongly discouraged to use different MRI equipment when performing a follow-up study.

Another technical element that can influence the final result in MRS studies is the type of spectral profile analysis. Most of the scientific reports have presented ratios of metabolites, especially referred to Cr, that is considered to be sufficiently stable. On the other hand, when managing large databases, it could be useful to perform a comparison with normative values via relevant software such as the “LcModel.” Ideally, although extremely time-consuming, absolute concentration analysis directly in acquisition could provide better comparison between the brain metabolites and the normal concentrations.

An important question concerns whether MRS has sufficient power to provide useful information on its own. The studies analyzed in this systematic review have limited power due to the small sample sizes, subsequently making it difficult to perform a meta-analysis. Only one study has reported the use of a single imaging technique in SPG4 with efficient differentiation of patients *versus* HC (20). The constant decrease of NAA peak and the mI increase, indexes of neuronal depletion and astrogliosis reaction as demonstrated by these papers, is of great interest. Therefore, we recommend that future MRS studies in HSP should investigate specifically NAA and mI. An HSP-phenotype related to TCC, could be further studied with BG and thalamus spectra for lipid detection, that highlights the possibility that a combination of MRI techniques could provide simultaneous structural and functional information.

Important open questions concern the applicability of MRS in the everyday clinical practice. Should MRS be used in the diagnostic process? We believe that there is not enough evidence to advice MRS as an additional tool in the diagnostic process. Can we rely on MRS findings to detect pre-symptomatic HSP? Yes, MRS could deepen the insight of early-stage disease. Could MRS be useful in predicting the disease progression or providing clinical trial biomarkers? MRS could be considered useful for the diagnostic process and perhaps monitoring the disease progression and response to treatment, as suggested in its general application (1). Is it wise to consider it a complementary technique to morphometry/DTI or a second-level imaging tool? MRS data alone, are not sufficient to define the clinical pictures. Therefore, MRS has absolutely a complementary role with morphometry/DTI techniques, and altogether could enrich the HSP picture with multiple information gained from the diagnostic work-up, clinical trials or treatment efficacy.

MRS studies have provided important insights in other CNS disorders. Hadjivassiliou et al. (26) presented evidence of the MRS usefulness in studying Gluten Ataxia. A follow-up study in spinocerebellar ataxias (SCAs) and multi-system atrophy denotes metabolites variation over time, but also questions their feasibility as disease progression biomarkers (27). Another study (28) has established the feasibility of detecting neurochemical changes in the brain in SCA carriers in the preclinical stage.

MRS acquisition in the spinal cord has significant technical difficulties, but it has so far advanced the knowledge regarding the metabolite concentrations in the cervical cord and the comparison with brain-stem metabolite patterns (29). The utility of spinal cord MRS indexes as disease biomarker(s) still needs to be confirmed.

## Conclusions

This systematic review provides a general overview of the state-of-the-art of brain metabolites in HSP. These results could help in designing hypothesis-driven clinical trials in HSP. Future studies should include longitudinal designs and bigger sample sizes, possibly pooling resources and multicentric studies. A specific disease severity measure should be always included in order to explore and understand the correlation with clinical status and disease progression. We recommend to study absolute metabolite concentrations, 3.0 T MRI equipment scan, and specifically cortical-subcortical WM samplings along the CST regions. It is fundamental to take into account the NAA and mI metabolites as sensitive to disease conditions underneath a HSP brain. The detection of the lipid peaks could add to a further specific differential diagnosis. Under a more specific technical point of view, short TE and single voxels acquisitions appear the more robust to select and measures each main metabolites (NAA, Cho, Cr, Lip, Lac). The search for new specific metabolites within the MRS spectrum could be driven by new data from genetic and biochemical studies. Importantly, the various types of follow-up studies should be applying the same identical methods, MRI equipment scan, anatomic localizations, and technical sequences of acquisition.

A future perspective sits with the creation of international consortia collecting large case series with harmonized methodology and the adoption of sound research criteria. We hope this review will encourage further steps in this direction.

## Author Contributions

MV and DM contributed to the conception and design of the review and contributed to the analysis. MV, DM, FF, and SP contributed to the literature search and contributed to the creation of tables and figures. MV and DM contributed to the data collection. MV contributed to the writing of the manuscript. MV, DM, SP, FF, FA, AB, GPi, GPa, and AM contributed to the critical review of the manuscript. All authors contributed to the article and approved the submitted version.

## Conflict of Interest

The authors declare that the research was conducted in the absence of any commercial or financial relationships that could be construed as a potential conflict of interest.
