# Supplementary material for: Brain Magnetic Spectroscopy Imaging and Hereditary Spastic Paraplegia: A Focused Systematic Review on Current Landmarks and Future Perspectives
Source: Front Neurol. 2020 Jul 14;11:515. doi: 10.3389/fneur.2020.00515 (PMC7381200; doi:10.3389/fneur.2020.00515)
Supplement: Supplementary file 1 [file Data_Sheet_1.pdf]

SUPPLEMENTARY METHODS

Search strategy

The authors (MV, DM) performed a comprehensive computer-based literature search on the databases PubMed MEDLINE, Cochrane Library and Embase in order to find studies relevant to the eligibility criteria for this systematic review on the brain MRS in HSP.

The search algorithm included the following keywords “HEREDITARY SPASTIC PARAPLEGIA; HEREDITARY SPASTIC PARAPARESIS; MR SPECTROSCOPY” and included all the items published until 21st July 2019. We screened the references of the retrieved articles to search for further relevant publications.

The outcome of the literature search is reported in the flowchart in Supplementary Figure 1. After screening the 169 records, 121 records were excluded as they were reviews, minireviews, thesis, conference proceedings and duplicates. Twenty-one articles were considered as full-text articles for eligibility. Additional 7 articles were excluded according to the non compliance with the review selection criteria due to absence of HSP genetic diagnosis of the patients studied or for other diagnosis different from HSP. Finally, only 14 articles completely satisfied the inclusion criteria and were analyzed for the qualitative synthesis.

Eligibility criteria

The studies considered for this systematic review reported original researches in patients with confirmed clinical and genetic diagnosis of HSP, who underwent brain spectroscopy neuroimaging, including case reports. The longitudinal original research studies were considered eligible and included for this review. We excluded review papers, conference proceedings, thesis, comments, editorials or letters. We excluded any other diagnosis. Only papers in English were considered for this review.

Data collection process

After the final selection of the papers eligible for the systematic review, elements were extracted in order to provide for each paper the following information: reference, year of publication, journal, first author's country and affiliation, type of study, genetic diagnosis, patients' group size, healthy control (HC) group size, age at the MRS scan of participants, disease duration, age at onset, disease severity measures, other clinical measures, pharmacological treatments. For each report were defined: scan properties (field strength, methodological features), brain areas investigated, if sampled specifically white (WM) or grey matter (GM) areas, levels of NAA, Cho, Creatine, mI, lipids, Lac, correlations between clinical measures, other neuroimaging techniques applied in addition to MRS, main results, novelties/advantages, critics/limitations reported. The characteristics of the included studies are presented in the main text in Tables 1 and 2. The quality of each study was assessed by two different and specific tools for case report and case series (The Carmen Moga and his colleagues' tool) (1) and for case- control studies (NICE methodology checklist for case-control study, (NICE) (2). The global quality of studies was explored by using *Moga and Nice tools* and the results are reported as graph presentation in Supplementary Figure 2A and 2B.

Summary measures and Synthesis of results

All the variables considered were collected in a unique table. Subsequently, the data were read and analyzed according to the following main points: demographic characteristics of the patients, technical aspects of the MRS scans and MRS findings.

Furthermore, we analyzed the quality of each study and explored whether there was a trend in the MRS findings according to the SPG subtypes.

Considering the small number of the studies included for the systematic review, the discrepancy in the type of results presented and of the methodology, no quantitative analysis was performed.

SUPPLEMENTARY RESULTS

Clinical and instrumental outcome measures

All records reported that patients had been assessed with neurological examination. In particular, 3 studies (3, 4, 5) reported data on disease severity assessed with the specific HSP scale known as Spastic Paraplegia Rating Scale (SPRS) (7), one study used the landmarks of disability (8) and another used a disability score (9). Other specific motor outcome measures used were Timed Up and Go test (4), 6-minute walking test, muscle strength with Medical Research Council measure and Functional Independence Measure (3).

The cognitive and neuropsychological profile was generally explored with intellectual quotient measures (5, 6, 10), Mini Mental Status Examination (6, 8, 10, 11), ENB-2 (3) and neuropsychological battery (12). In particular, Erichsen et al. (9) explored the cognitive functions by assessing attention, memory, the psychomotor speed, and executive functions.

Most of the studies performed neurophysiological examinations such as electroencephalography, nerve conduction studies, electromyography, evoked potentials (somatosensory, motor, visual), optical coherence tomography and transcranial sonography.

Quality of studies (Supplementary Figure 2a, b)

By using the MOGA tool we analyzed the quality of the case reports and case series (Figure 2a). Mainly, these studies presented a very good description of the participants' characteristics, outcome measure methods, conclusions, competing interests and funding information.

By using the NICE tool, we analyzed the quality of the case controls (Figure 2b). In these cases, the included studies accurately differentiated the experimental and control groups, presented comparable populations and addressed a clearly focused research question.

SUPPLEMENTARY REFERENCES

1. Moga C, Guo B, Schopflocher D, Harstall C. Development of a Quality Appraisal Tool for Case Series Studies using a Modified Delphi Technique. 2012. http://www.ihe.ca/documents/Case%20series%20studies%20using%20a%20modified%20Delphi%20technique.pdf Accessed in June, 2014.
2. NICE; http://www.nice.org.uk/
3. Martinuzzi A, Montanaro D, Vavla M, Paparella G, Bonanni P, Musumeci O, Brighina E, Hlavata H, Rossi G, Aghakhanyan G, Martino N, Baratto A, D'Angelo MG, Peruch F, Fantin M, Arnoldi A, Citterio A, Vantaggiato C, Rizzo V, Toscano A, Bresolin N, Bassi MT. Clinical and Paraclinical Indicators of Motor System Impairment in Hereditary Spastic Paraplegia: A Pilot Study. PLoS One. 2016 Apr 14;11(4): e0153283. doi: 10.1371/journal.pone.0153283. ECollection 2016. PMID: 27077743.
4. Schneider-Gold C, Dekomien G, Regensburger M, Schneider R, Trampe N, Krogias C, Lukas C, Bellenberg B. Monozygotic twins with a new compound heterozygous SPG11 mutation and different disease expression. J Neurol Sci. 2017 Oct 15; 381:265-268. doi: 10.1016/j.jns.2017.09.005. Epub 2017 Sep 5.
5. Nicita F, Stregapede F, Tessa A, Bassi MT, Jezela-Stanek A, Primiano G, Pizzuti A, Barghigiani M, Nardella M, Zanni G, Servidei S, Astrea G, Panzeri E, Maghini C, Losito L, Ploski R, Gasperowicz P, Santorelli FM, Bertini E, Travaglini L. Defining the clinical-genetic and neuroradiological features in SPG54: description of eight additional cases and nine novel DDHD2 variants. J Neurol. 2019 Jul 13. doi: 10.1007/s00415-019-09466-y. [Epub ahead of print] PMID: 31302745.
6. Lossos A, Elazar N, Lerer I, Schueler-Furman O, Fellig Y, Glick B, Zimmerman BE, Azulay H, Dotan S, Goldberg S, Gomori JM, Ponger P, Newman JP, Marreed H, Steck AJ, Schaeren-Wiemers N, Mor N, Harel M, Geiger T, Eshed-Eisenbach Y, Meiner V, Peles E. Myelin-associated glycoprotein gene mutation causes Pelizaeus-Merzbacher disease-like

disorder. Brain. 2015 Sep;138(Pt 9):2521-36. doi: 10.1093/brain/awv204. Epub 2015 Jul 15. PMID: 26179919.

7. Schüle R, Holland-Letz T, Klimpe S, Kassubek J, Klopstock T, Mall V, Otto S, Winner B, Schöls L. The Spastic Paraplegia Rating Scale (SPRS): a reliable and valid measure of disease severity. Neurology. 2006 Aug 8;67(3):430-4. PMID: 16894103.
8. Stromillo ML, Malandrini A, Dotti MT, Battaglini M, Borgogni F, Tessa A, Storti E, Denora PS, Santorelli FM, Gaudiano C, Battisti C, Federico A, De Stefano N. Structural and metabolic damage in brains of patients with SPG11-related spastic paraplegia as detected by quantitative MRI. J Neurol. 2011 Dec;258(12):2240-7. doi: 10.1007/s00415-011-6106-x. Epub 2011 May 29.
9. Erichsen AK, Server A, Landrø NI, Sandvik L, Tallaksen CM. Proton magnetic resonance spectroscopy and cognition in patients with spastin mutations. J Neurol Sci. 2009 Feb 15;277(1-2):124-9. doi: 10.1016/j.jns.2008.10.030. Epub 2008 Dec 12. PMID: 19084842.
10. Fraidakis MJ, Brunetti M, Blackstone C, Filippi M, Chiò A. Novel Compound Heterozygous Spatacsin Mutations in a Greek Kindred with Hereditary Spastic Paraplegia SPG11 and Dementia. Neurodegener Dis. 2016;16(5-6):373-81. doi: 10.1159/000444715. Epub 2016 Jun 18.
11. Liguori R, Giannoccaro MP, Arnoldi A, Citterio A, Tonon C, Lodi R, Bresolin N, Bassi MT. Impairment of brain and muscle energy metabolism detected by magnetic resonance spectroscopy in hereditary spastic paraparesis type 28 patients with DDHD1 mutations. J Neurol. 2014 Sep;261(9):1789-93. doi: 10.1007/s00415-014-7418-4. Epub 2014 Jul 3. PMID: 24989667.
12. Svenstrup K, Giraud G, Boespflug-Tanguy O, Danielsen ER, Thomsen C, Rasmussen K, Law I, Vogel A, Stokholm J, Crone C, Hjermind LE, Nielsen JE. Hereditary spastic paraplegia caused by the PLP1 'rumpshaker mutation'. J Neurol Neurosurg Psychiatry. 2010 Jun;81(6):666-72. doi: 10.1136/jnnp.2009.180315. Epub 2009 Dec 1.

SUPPLEMENTARY FIGURE

Supplementary Figure 1. Flow chart of the search for eligible studies on the spectroscopy findings of the brain metabolites in Hereditary Spastic Paraplegia.

Supplementary Figure 2. Quality assessment in case series (MOGA tool) **(a)** and in case-control studies (NICE tool) **(b)**.
