# Supplementary figures and images for: Brain Magnetic Spectroscopy Imaging and Hereditary Spastic Paraplegia: A Focused Systematic Review on Current Landmarks and Future Perspectives
Source: Front Neurol. 2020 Jul 14;11:515. doi: 10.3389/fneur.2020.00515 (PMC7381200; doi:10.3389/fneur.2020.00515)

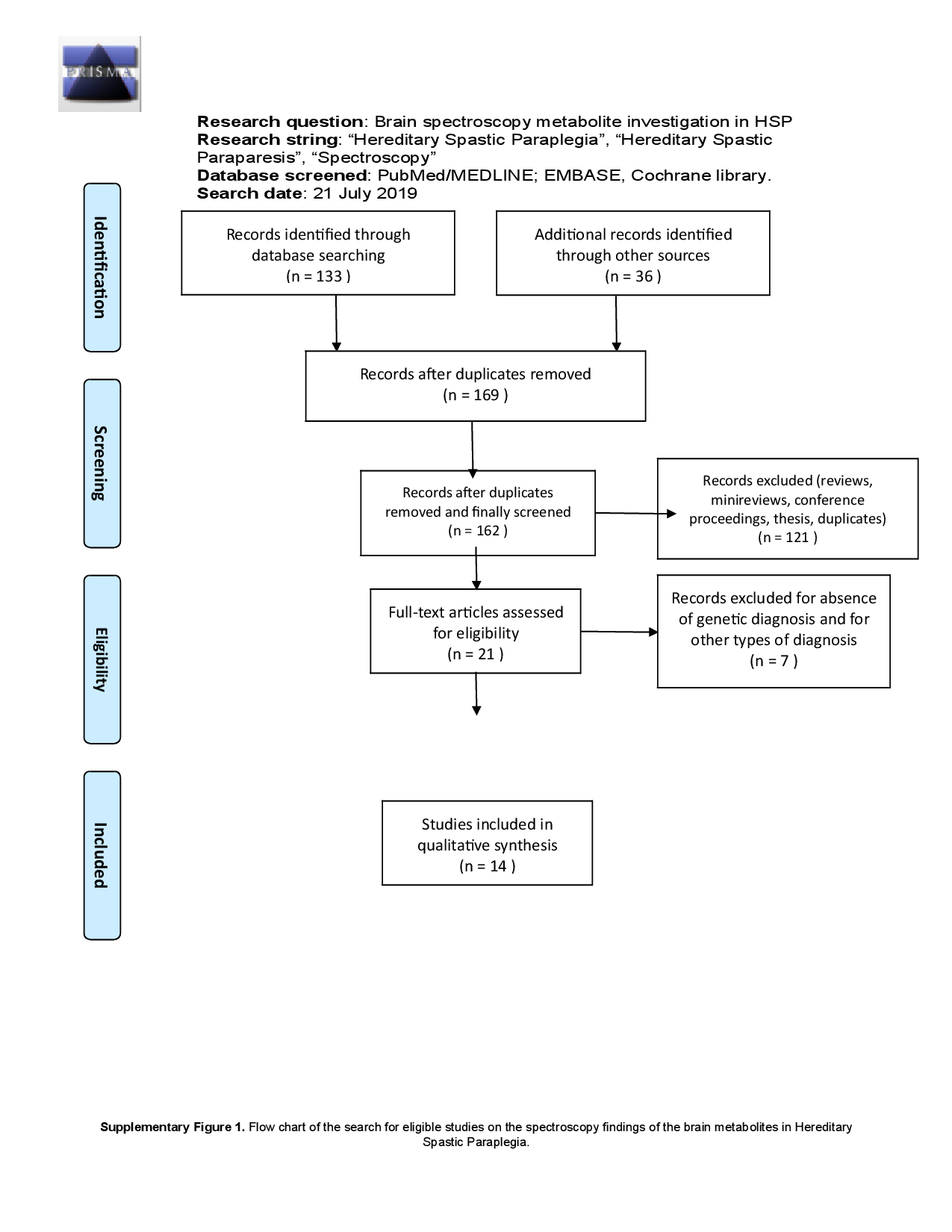

Supplement: Supplementary file 2 [file Image_1.TIF]

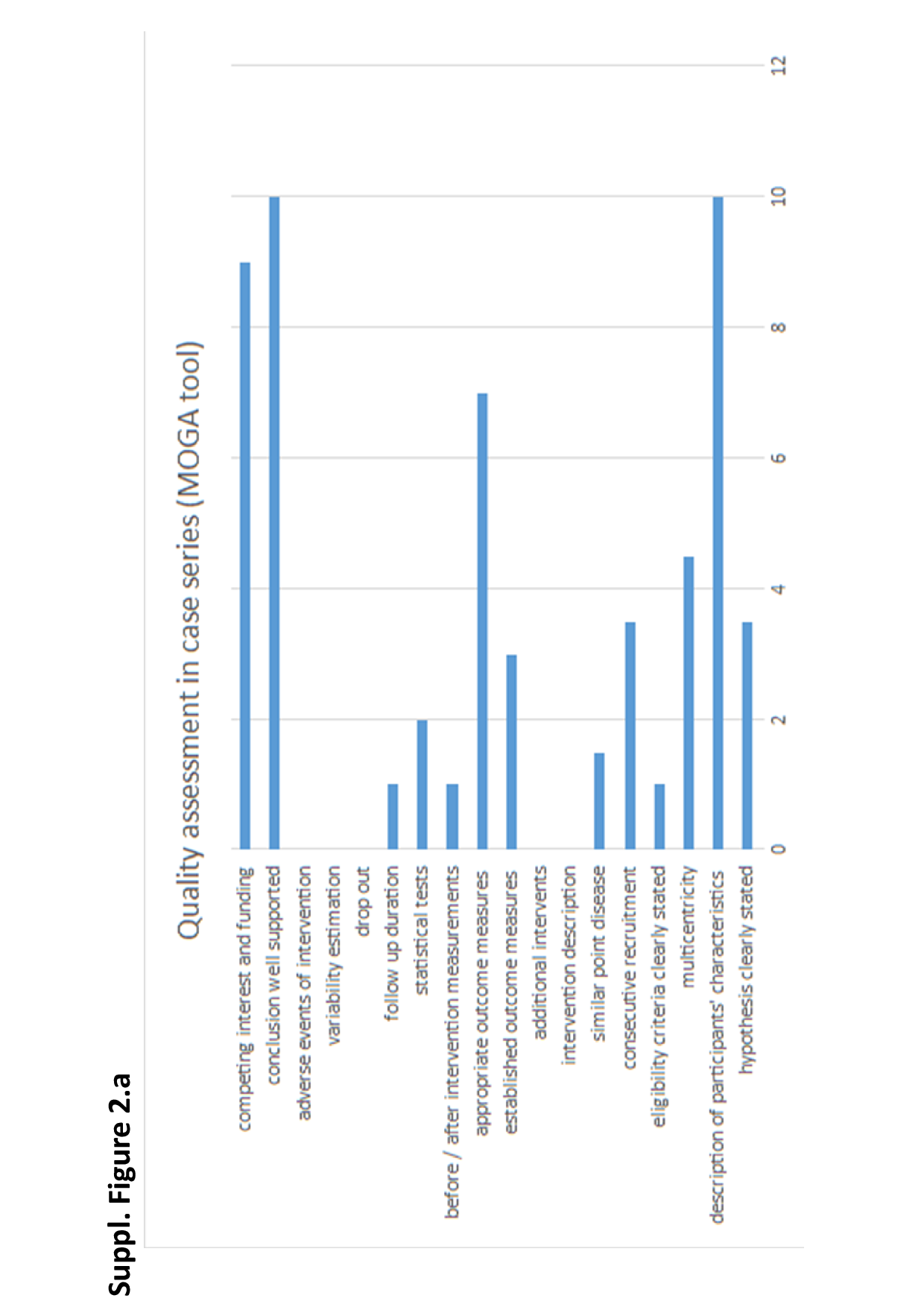

Supplement: Supplementary file 3 [file Image_2.TIF]
